# The Little Helper

**Published:** 1879-06

**Authors:** J. G. Harper

**Affiliations:** St. Louis


### THE LITTLE HELPER.

BY J. G. HARPER, D. D. S., ST. LOUIS.

The little instrument I wish to describe has been a great help to me, and I hope that all who read these few lines may feel the same pleasure that I experience when using it.

To make the *helper* take a piece of hard wood a quarter of an inch in diameter and about an inch in length, cut in each end a groove parallel one to the other. You now have the assistant, but do not know its uses. One of the places where it will help you is in finishing proximal fillings in the front teeth, with slips of sand paper or lava strips; the operator is greatly annoyed by the strips slipping off the end of the tooth; this can be prevented by the helper; after putting the strip between the teeth the helper is placed so that one end rests against the ends of the teeth, between which the sand paper has been placed, the cutting edges of the teeth occupying the groove; the other end is pressed against by the antagonizing teeth in the opposite jaw. Now take the ends of the slip between the thumb and fore finger of each hand and draw rapidly back and forth over the filling, cutting very rapidly until the filling is dressed down to the surface of the tooth, then turn the slip and let the back of the paper touch the filling; draw this a few times over the filling and you have a beautiful finish. Another place where the helper can be used, is when filling a tooth that is pained by the lateral blow of the mallet. Place the helper as described, and instruct the patient to keep the teeth pressed hard against the piece of wood. This holds the tooth firm and keeps it free from pain to the great satisfaction of both patient and operator.

